# Halogenated Indole Alkaloids from Marine Invertebrates

**DOI:** 10.3390/md8051526

**Published:** 2010-04-28

**Authors:** Patrícia Mendonça Pauletti, Lucas Silva Cintra, Caio Guedes Braguine, Ademar Alves da Silva Filho, Márcio Luís Andrade e Silva, Wilson Roberto Cunha, Ana Helena Januário

**Affiliations:** Núcleo de Pesquisas em Ciências Exatas e Tecnológicas, Universidade de Franca. Av. Dr. Armando Salles de Oliveira, 201, CEP 14404-600, Franca, São Paulo, Brazil; E-Mails: pmpauletti@unifran.br (P.M.P.); lscintra@uol.com.br (L.S.C.); caiobraguine@aluno.unifran.br (C.G.B.); ademar@unifran.br (A.A.S.F.); mlasilva@unifran.br (M.L.A.S.); wrcunha@unifran.br (W.R.C.)

**Keywords:** marine invertebrates, halogenated indole alkaloids, structure elucidation, bioactivity, ^13^C-NMR spectral data

## Abstract

This review discusses the isolation, structural elucidation, and biological activities of halogenated indole alkaloids obtained from marine invertebrates. Meridianins and related compounds (variolins, psammopemmins, and aplicyanins), as well as aplysinopsins and leptoclinidamines, are focused on. A compilation of the ^13^C-NMR spectral data of these selected natural indole alkaloids is also provided.

## 1. Introduction

Marine organisms are among the most promising sources of bioactive molecules [1–3]. Unlike terrestrial organisms, marine organisms often produce halogenated secondary metabolites, particularly alkaloids [4]. The majority of halogenated metabolites contain bromine and they are especially abundant in the marine environment, whereas chlorinated compounds are preferably synthesized by terrestrial organisms. In contrast to brominated and chlorinated metabolites, iodinated and fluorinated compounds are quite rare [5,6].

Marine life produces most of the 4,000 known natural organohalogens. Almost all of the 2,100 natural organobromine compounds are found in marine organisms. Although there is much less bromide than chloride in the oceans (bromine 65 mg/L; chlorine 19,000 mg/L), marine organisms can oxidize bromide more easily for incorporation into organic compounds. Nevertheless, a large number of marine metabolites contain both bromine and chlorine [7,8].

Ecological pressures within the marine ecosystem, including significant competition for space, deterrence of predation, and a high level of symbiosis between different species, are partially responsible for the unique secondary metabolism of marine life that give rise to the chemical components of these actions and interactions [9,10].

The presence of halogen substituents in many natural products profoundly influences their biological activity [6]. Examples of such biologically active compounds are the antibiotics vancomycin, chloramphenicol aureomycin, and salinosporamide A; a proteasome inhibitor currently under clinical trials for multiple myeoloma treatment; and the antimicrobial rebeccamycin [6,8].

Among halogenated alkaloids, bromoalkaloids are the most widely distributed group of natural compounds. This group is predominantly found in marine eukaryotes, is significantly rarer in prokaryotic microorganisms, and is practically absent from terrestrial plants and animals [4]. Iodoalkaloids compose a rare group of natural compounds that has been isolated from marine organisms [4].

**Figure 1:**
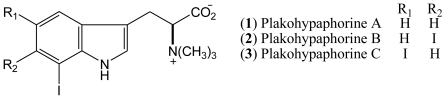
Structures of plakohypaphorines A, B, and C (**1**–**3**).

The first iodinated indoles found in a natural source, either marine or terrestrial, were the plakohypaphorines A–C (**1**–**3**, Figure 1) isolated from the Caribbean sponge *Plakortis simplex* [8,11].

A huge diversity of indole alkaloids are frequently found in marine invertebrates and they have been considered lead compounds for the discovery of new drugs in medicinal chemistry [9,12]. The biological activity of marine indole alkaloids is clearly a product of the unique functionality and elements involved in the biosynthesis of marine natural products. For instance, bromination of many natural products has the potential to increase biological activity significantly [9].

In this report we have focused on the halogenated indole alkaloids from marine invertebrates, particularly meridianins; their related compounds variolins, psammopemmins, and aplicyanins as well as the aplysinopsins and leptoclinidamines. Also summarized are the methods of structure determination, observed biological activities and a compilation of ^13^C-NMR spectral data is provided.

### 1.1. Biohalogenation

The halogenation of natural products is a frequent modification of secondary metabolism that allows for optimization of the bioactivity of small molecules, providing evolutionary advantage [6].

Many biohalogenation enzymes have been isolated and characterized. Chloroperoxidase, bromoperoxidase, iodoperoxidase, and the enzymes involved in the biosynthesis of fluoroacetic acid (fluoroacetaldehyde dehydrogenase and 5′-fluorodeoxyadenosine synthase) are some examples [8].

Halogenating enzymes have been discovered in a broad range of organisms and they can be grouped into two main classes: (i) highly substrate-specific halogenases requiring dioxygen for enzymatic activity and (ii) less specific haloperoxidases (HPO) utilizing hydrogen peroxide. In dioxygen-dependent halogenases, either flavin (FADH_2_-dependent halogenases) or R-ketoglutarate (non-heme FeII/R-ketoglutarate/O_2_- dependent halogenases) are found to function as co-substrates. Furthermore, methyltransferases are involved in the formation of the carbon halogen bonds of CH_3_Cl, CH_3_Br, and CH_3_I, and other enzymes requiring *S*-adenosyl-l-methionine as catalyst have been identified to be involved in fluorination and chlorination [13].

In the recent years, the understanding of biohalogenation processes has been extended extraordinarily. The cloning and sequencing of biosynthetic gene clusters have revealed new mechanisms leading to halogen incorporation and stimulated detailed mechanistic studies of these enzymes [6,8]. New groups of halogenating enzymes have been discovered and investigated at both biochemical and genetic levels. Each group of these enzymes performs halogenation reactions on chemically distinct substructures using a specific reaction mechanism. For instance, some FADH_2_-dependent halogenases are directly involved in the halogenation of aromatic compounds, recognizing tryptophan or indole moieties, while other groups of FADH_2_-dependent halogenases participate in the halogenation of aliphatic compounds [13].

### 1.2. Meridianins

**Figure 2:**
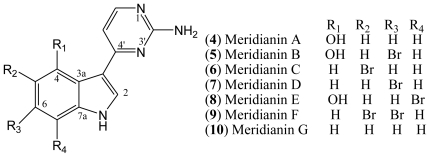
Structures of meridianins **4**–**10**.

Meridianins are marine alkaloids which were first isolated from the Ascidian *Aplidium meridianum* [14]. Structurally, the meridianins comprise a brominated and/or hydroxylated indole nucleus substituted at C-3 by a 2-aminopyrimidine. Seven meridianins A–G (**4**–**10**) have been discovered so far. Bromine substitution occurs on position 5 for meridianin C (**6**), on position 6 for B (**5**) and D (**7**), on position 7 for E (**8**), and on positions 5 and 6 for F (**9**) (Figure 2).

Meridianins have been described as potent inhibitors of various protein kinases (Table 1) and they display antitumor activity. Meridianins B (**5**) and E (**8**) are the most potent and, for this reason meridianin E was selected for further selectivity studies on 25 highly purified kinases [15]. Essentially, all physiological processes and most human diseases involve protein phosphorylation. Phosphorylation of proteins on serine, threonine, and tyrosine residues by the 518 protein kinases encoded in the human genome constitutes one of the major mechanisms used by cells to regulate their metabolism and functions. The recent appreciation of the implication of abnormal protein phosphorylation in many human diseases has sparked considerable interest in the search for pharmacological inhibitors of kinases [16–18].

Protein phosphorylation regulates most aspects of cell life, whereas abnormal phosphorylation is a cause or consequence of diseases. For instance, among the 518 human kinases cyclin-dependent kinases (CDK) have attracted considerable interest given their involvement in many essential physiological pathways and numerous abnormalities in multiple human diseases, especially cancer and neurodegenerative diseases such as Alzheimer’s and Parkison’s diseases [16,18,19].

Investigations of structure-activity relationships of meridianins have revealed that the substitution at C-5 and the methylation of the indole nitrogen are important for either kinase inhibitory activity or *in vitro* antiproliferative activities. Related to CDK1 and CDK5, the bromine substitution on position 7 and the hydroxyl on position 4 provide the best inhibitory activity. A single bromine substitution on position 5 or 6 of the indole ring results in considerable improvement in potency. On the other hand, two bromide substitutions slightly reduce the inhibitory potency [20,21].

Meridianins B, C, D, and E (**5**–**8**) display cytotoxicity toward LMM3 (murine mammalian adenocarcinoma cell line) with IC_50_ values of 11.4 μM, 9.3 μM, 33.9 μM, and 11.1 μM, respectively [14]. Certainly, meridianins constitute a new scaffold exhibiting micromolar inhibition of protein kinases from which more potent and selective inhibitors can be designed [15].

Meridianins are closely related to the variolins, a class of marine alkaloids from the Antarctic sponge *Kirkpatrickia varialosa* [22,23].

### 1.3. Variolins

In 1994, the Blunt, Munro and Faulkner laboratories reported the isolation and structural elucidation of the variolins from the rare Antarctic sponge *Kirkpatrickia varialosa* [22,23]. Variolins are the first examples of either terrestrial or marine natural products with a pyrido[3′,2′:4,5]pyrrolo[1,2-c]pyrimidine system. This rare pyridopyrrolopyrimidine skeleton has made the variolins an interesting class of alkaloids from both structural and biogenetic viewpoints. Variolins can also be considered as guanidine-based alkaloids in which the guanidine moiety is found in the guise of a 2-aminopyrimidine ring [24–26].

**Figure 3:**
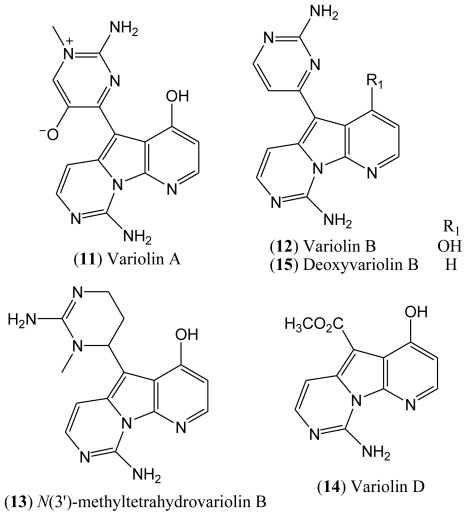
Structures of variolins **11**–**15**.

The isolated compounds included variolin A (**11**), variolin B (**12**), *N*(3′)-methyl tetrahydrovariolin B (**13**), and variolin D (**14**), the latter of which was reported to be an artifact of the extraction process produced by aerial oxidation of the variolins (Figure 3). This type of compounds exhibit a potent cytotoxic activity against P388 murine leukemia cell line, also being effective against Herpes simplex type I. Variolin B (**12**) is the most active of this family of natural products [26].

There has been considerable interest in the synthesis of variolins due to the novelty of their structures, not to mention their biological properties and low natural occurrence [25]. To date, four total syntheses of variolin B have been reported in the literature [21,27–33], and the preparation of the synthetic deoxyvariolin B (**15**) has also been described [34,35]. The synthesis of new derivatives of variolin B with different substituents at positions C-5 and C-7 has also been reported [26].

**Figure 4:**
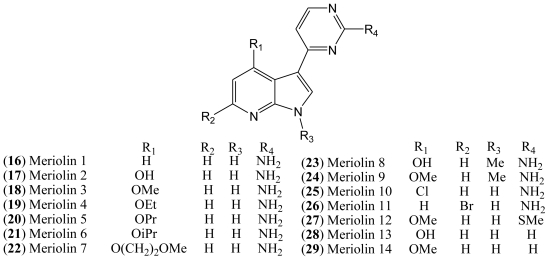
Structures of meriolins **16**–**29**.

Although the natural variolins isolated are not halogenated, this type of skeleton along with the structure of meridianins have been an inspiration for the synthesis of the hybrid meriolins 1–14 (**16**–**29**, Figure 4), including the halogenated meriolins 10 (**25**) and 11 (**26**) [18].

### 1.4. Meriolins

Variolins with a pyridopyrrolopyrimidine system and meridianins possessing a pyrimidyl-substituted indole skeleton bear some structural similarities. Through a combination of the common features of these natural products, a new class of 7-azaindole-containing analogues (**16**–**29**) known as meriolins has been designed by Meijer and co-workers [21].

Meriolins [3-(pyrimidin-4-yl)-7-azaindoles], a chemical hybrid of the variolins and meridianins, display potent inhibitory activity toward CDKs (especially CDK2 and CDK9). This class of compounds also exhibit better antiproliferative and proapoptotic properties in cell cultures compared with their “inspirational parent” molecules [18,19].

The resemblance between the chemical structures of the two natural products meridianins and variolin B has inspired the synthesis of a hybrid structure referred to as meriolins, which display better antiproliferative and proapoptotic properties in human tumor cell cultures than their parent molecules. A selectivity study performed on 32 kinases has shown that, compared with variolin B, meriolins exhibit enhanced specificity toward CDKs, with marked potency on CDK2 and CDK9 [19].

The structures of pCDK2/cyclin A/meriolin 3, pCDK2/cyclin A/meriolin 5, and pCDK2/cyclin A/variolin B complexes have been determined by X-ray crystallography, which revealed that these inhibitors bind within the ATP binding site of the kinase, but in different orientations [18,19,21].

All synthesized meriolins 1–14, along with variolin B as a reference, were tested on seven purified protein kinases, namely CDK1/cyclin B, CDK2/cyclin A, CDK5/p25, CDK9/cyclin T, GSK-3 δ/β, CK1δ/ɛ, and DYRK1A (Table 2). Structure-Activity studies complemented with the crystal structure have provided some clarification on the action mechanisms of these molecules on their CDK target [18].

In the case of meriolin 11 (**26**), addition of a bromide atom at C-5 leads to a drop in inhibitory activity for almost all tested protein kinases, but this effect is particularly pronounced against CDK9 and GSK-3. CDK1, CDK2, and CDK5 are less affected by the bromide addition. Moreover, addition of a chloride atom at C-4 in meriolin 10 (**25**) results in decreased potency compared to the non-halogenated meriolin 1 (**16**). Taken together, these observations suggest that meriolins constitute a new CDK inhibitory scaffold with promising antitumor activity, and they can be derived from molecules initially isolated from marine organisms [19].

### 1.5. Psammopemmins

**Figure 5:**
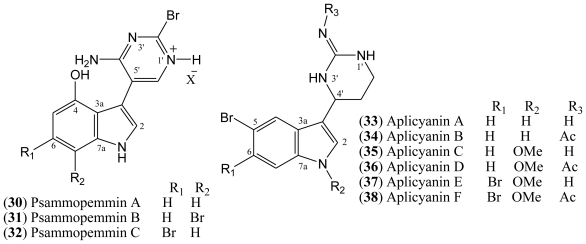
Structures of psammopemmins **30**–**32** and aplycianins **33**–**38**.

Psammopemmins represent an unusual group of natural products isolated as an amine salt from an Antarctic marine sponge *Psammopemma* sp and they comprise three structurally related compounds designated psammopemmins A–C (**30**–**32**, Figure 5). All of the psammopemmins incorporate the 4-hydroxyindole moiety substituted at the 3-position by an unusual 2-bromopyrimidine system. Compounds containing 4-oxygenated indoles often display potent pharmacological properties. Psammopemmins B (**31**) and C (**32**) contain further bromination on the indole ring. Unfortunately, the small amounts of material isolated so far have precluded any further investigation of their biological activity. The assigned structure of the psammopemmin family likewise remains to be confirmed by total synthesis [21,36].

### 1.6. Aplicyanins

A new family of indole alkaloids was recently isolated from the Antarctic tunicate *Aplidium cyaneum* by Reyes and co-workers [37]. The aplicyanins A–F (**33**–**38**, Figure 5) contain a bromoindole nucleus and a 6-tetrahydropyrimidine substituent at C-3. The main structural variations present in aplicyanins include additional bromination of indole ring and the presence of *N*-methoxy group as shown in aplicyanins C–F (**35**–**38**). The aplicyanins share a common 3-(pyrimid-4-yl)indole structure with meridianins A–G (**4**–**10**), the psammopemmins A–C (**30**–**32**) and variolins A–D (**11**–**14**). The tetrahydropyrimidine system of the aplicyanins has a stereocenter at C 4′, in contrast with the planar pyrimidine ring of the meridianins [21].

Aplycianins are cytotoxic to the human tumor cell lines MDA-MB-231 (breast adenocarcinoma), A549 (lung carcinoma), and HT-29 (colorectal carcinoma). They also exhibit antimitotic activity [38]. Lastly, given the high cytotoxicity typical of bromoindole derivatives, the presence of a bromoindole moiety in some aplicyanins warrants their investigation as anticancer drugs. Recently, the first total synthesis of (±)-aplicyanins A, B, and E and 17 analogues has been reported [38].

Regarding the aplicyanin family of indole alkaloids, the six variants of aplicyanins isolated were evaluated for cytotoxicity against a panel of three human tumor cell lines, colon (HT-29), lung (A-549), and breast (MDA-MB-231). The antimitotic activity of these variants has also been assessed. Cytotoxic activity in the submicromolar range as well as antimitotic properties have been found for aplicyanin B (**34**), D (**36**), and F (**38**), with IC_50_ values in the low to sub-μM range. On the other hand, aplicyanin A (**33**) and C (**35**) proved to be inactive at the highest concentrations tested, whereas aplicyanin E (**37**) displayed weak cytotoxic properties (Table 3). These results indicate a key role for the presence of the acetyl group in the biological activity of the aplicyanin family [37].

In order to establish the structure-activity relationships of the aplicyanins, the total synthesis of (±)-aplicyanins A, B, and E, plus 17 analogues was carried out by Sísa and co-workers in 2009 [38]. The compounds were again screened for cytotoxicity against the same three human tumor cell lines used for the natural compounds. Racemic (±)-aplicyanin A exhibited activity in the submicromolar range, despite the inactivity of the corresponding natural product. Racemic (±)-aplicyanin B was as active as its corresponding natural product in all three tested cellular lines, whereas aplicyanin E maintained the activity only towards the MDA-MB-231cell line (Table 3). The decreased cytotoxicity observed for racemic aplicyanin E compared to the natural product, indicates that one enantiomer is more active than the other [38].

Fourteen of the synthesized compounds also exhibited considerable cytotoxic activity, and these results suggest that the bromine at position 5 of the indole nucleus strongly favors antiproliferative activity, and the acetyl group at the imine nitrogen also acts in some compounds. These results demonstrate the potential of aplycianins structure as a scaffold for anticancer drug discovery [38].

### 1.7. Aplysinopsins

In 1977, Kazlauskas, Rymantas, and co-workers reported the isolation of aplysinopsin (**39**) from the dictoyoceratid sponge *Aplysinopsis* [39,40]. Aplysinopsin derivatives belong to a class of indole alkaloids and they have also been found in other dictyoceratid and astrophorid sponges as well as in dendrophylliid scleractinian corals [41]. Additionally, aplysinopsins have been described in anemone, in a symbiotic association, and in a mollusk that feeds on the coral *Tubastrea coccinea* [39].

**Figure 6:**
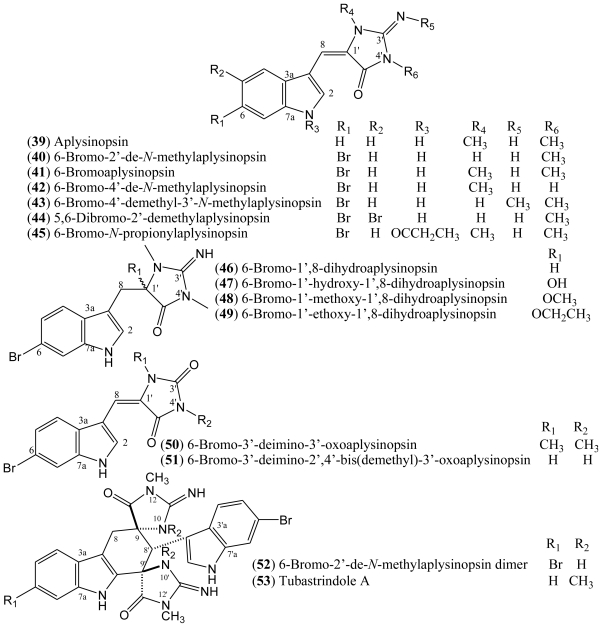
Structures of aplysinopsins **39**–**53**.

The halogenated aplysinopsins natural derivatives (Figure 6) contain a 6-bromoindole moiety, and an iminoimidazolidinone or imidazolidinedione system, both varying in terms of the number and position of *N*-methylation. The iminoimidazolidinone portion of compounds **39**–**45** are shown as the exocyclic imino tautomer. Only compound **44** contains an additional bromine at the C-5 of the indole core. The aplysinopsins derivatives also differ in terms of the presence and absence of the C-8-C-1′ double bond. Thus, aplysinopsins with C-8-C-1′ double bonds, the most abundant type, can occur as two geometrical isomers (*E*/*Z*). Also, it has been observed that (*Z*)-aplysinopsins are generally less abundant than the (*E*)-isomers [41,42]. Aplysinopsins substituted at the nitrogen atom of the indole ring and dimers have also been isolated or identified, although compound **45** could be an artifact [43–45].

Aplysinopsins exhibit cytoxicity towards tumour cells, as well as some antimalarial and antimicrobial activities. However, properties related to neurotransmission modulation seem to be the most significant pharmacological feature of these compounds. Aplysinopsins have the potential to influence monoaminooxidase (MAO) and nitric oxide synthase (NOS) activities. They have also been found to modulate serotonin receptors [39].

Aplysinopsin-type compounds have been reported from multiple sources, with brominated aplysinopsins being described from sponges [46–49], corals [41–45], anemone, and mollusks [50,51]. Natural aplysinopsins differ in the bromination pattern of the indole ring. Almost all natural occurring aplysinopsins display halogenations at the position 6 of the indole ring. The only exception is the compound 5,6-dibromo-2′-demethylaplysinopsin (**44**), which an additional bromine atom at C-5 [39].

The compounds 6-bromo-2′-de-*N*-methylaplysinopsin (**40**) and 6-bromoaplysinopsin (**41**) isolated from the Jamaican sponge *Smenospongia aurea* displayed high-affinity [^3^H]antagonist binding from cloned human serotonin 5-HT_2C_ receptors expressed in a mammalian cell line (*K_i_* = 2.3 μM and *K_i_* = 0.33 μM, respectively). Compound **41** also displayed high-affinity [^3^H]antagonist binding from the 5-HT_2A_ receptor subtype (*K_i_* = 2.0 μM) compared with serotonin affinity values *K_i_* = 0.32 μM at the 5-HT_2A_ receptor and *K_i_* = 0.13 μM at the 5-HT_2C_ receptor [46].

The structure-activity relationship data reveal a role for the R_1_, R_2_, and R_3_ functional groups at positions 6, 2′, and 3′, respectively, in the binding to human serotonin 5-HT_2_ receptors. The length of the alkyl chain at the R_3_ position as well as the bromination at position R_1_ seems to be important for activity. In addition, bromination at the R_1_ position is also relevant for the binding affinity of aplysinopsins and for their selective binding to the 5-HT_2C_ receptor subtype, since both compounds **40** and **41** are brominated and both selectively bind the 5-HT_2C_ receptor subtype over the 5-HT_2A_ receptor subtype. Methylation at the R_2_ position facilitates binding to the 5-HT_2A_ receptor subtype. A larger number of analogues will be required to confirm this proposed structure-activity relationship [46]. Pharmacological and genetic studies have revealed that these receptors influence feeding, glucose homeostasis, and the energy efficiency of physical activity, sleep, sensory processing and learning, affective functioning, and the pathophysiology of several neuropsychiatric disorders [52,53].

6-Bromo-2′-de-*N*-methylaplysinopsin (**40**) and 6-bromoaplysinopsin (**41**) have also been tested *in vitro* against a D6 clone of *Plasmodium falciparum* for their *in vitro* antimalarial activity. 6-Bromoaplysinopsin (**41**) exhibited activity at 0.34 μg/mL with selective index 14 (S.I. = [IC_50_ (Vero cells)/IC_50_ (*P. falciparum*)], while 6-bromo-2′-de-*N*-methylaplysinopsin (**40**) showed moderate activity at 1.1 μg/mL with low selectivity. Moreover, compound **40** inhibited the antimalarial target plasmepsin II enzyme with IC_50_ 53 μM (FRET) and 66 μM (FP) [46].

Additionally, 6-bromoaplysinopsin (**41**) has been reported to be involved in the symbiotic association between *Radianthus kuekentbali* (sea anemone) and *Amphiprum perideraion* (anemone fish) [50].

A number of aplysinopsin alkaloids have also been evaluated for their neuromodulatory activity in two types of nitric oxide synthase (NOS) isozymes. Nitric oxide (NO) is known to be an important second messenger having numerous functions which regulate many physiological processes; e.g., inflammation, blood pressure regulation, platelet adhesion, neurotransmission, and defense mechanisms. The biosynthesis of NO is catalyzed by nitric oxide synthase (NOS), which is classified into three isoforms: inducible NOS (iNOS), endothelial NOS (eNOS), and neuronal NOS (nNOS). Therefore, a selective inhibitor of NOS isozymes would be expected to have significant therapeutic potential as a neuromodulator [47]. 6-Bromo-2′-de-*N*-methylaplysinopsin (**40**) and the isomers 5,6-dibromo-2′-demethylaplysinopsin *Z*-**44** and *E*-**44** isolated from the sponge *Hyrtios erecta* display selective inhibitory activity against nNOS, with 100% inhibition of nNOS at 125 μg/mL [47]. Compounds *Z*-**44** and *E*-**44** showed no inhibitory activity against iNOS. In turn, aplysinopsin **40** inhibited only 7.5% of iNOS activity at a concentration of 125 μg/mL [47].

Regarding the antimicrobial potential of halogenated aplysinopsins, Koh and Sweatman [54] have reported the screening of the Australian coral *Tubastraea faulkneri* extract for antimicrobial assay against seven species of microbes (*Vibrio alginolyticus*, *V. harveyi*, *V. parahaemolyticus*, *Photobacterium damsela*, *Alteromonas rubra*, *Staphylococcus aureus*, and *Synechococcus* sp). Aplysinopsin (**39**), 6-bromoaplysinopsin (**41**), 6-bromo-2′-de-*N*-methylaplysinopsin (**40**), and its dimer **52** were the compounds isolated accounting for 72% of the activity of the *T. faulkneri* methanol extract. This study also suggested that these aplysinopsins are toxic to the larvae of other coral species that are potential competitors and could act as allelochemicals [54]. The aplysinopsins **46**–**49** isolated from the sponge *Thorectandra* sp were evaluated for antimicrobial activity against *Staphylococcus epidermidi*. All of the compounds were found to have either weak or moderate minimum inhibitory concentrations (MIC) ranging from 6.25 to 100 μg/mL as compared to the standard vancomycin (0.625 μg/mL) [48].

### 1.8. Leptoclinidamines

**Figure 7:**
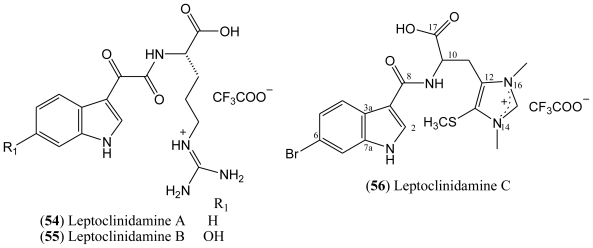
Structures of leptoclinidamines **54**–**56**.

Three new indole alkaloids, namely leptoclinidamines A–C (**54**–**56**, Figure 7), have been recently isolated from the Australian ascidian *Leptoclinides durus* [55].

The leptoclinidamines A (**54**) and B (**55**) both contain an indoleglyoxylic acid attached to an l-arginine residue, while leptoclinidamine C (**56**) contains the rare 1,3-dimethyl-5-(methylthio)histidine moiety attached to a 6-bromoindole-3-carboxylic acid. The structure of leptoclinidamine A was confirmed by total synthesis. The compounds were tested for bioactivity against chloroquine-sensitive and chloroquine-resistant strains of the malarial parasite *Plasmodium falciparum*, for trypanosomal activity against *Trypanosoma brucei*, and for cytotoxicity against the cancerous cell line HeLa and noncancerous HEK 293 cells, but none of the compounds were bioactive [55].

### 1.9. Chartelline, Chartellamide, Securamines and Securines

**Figure 8:**
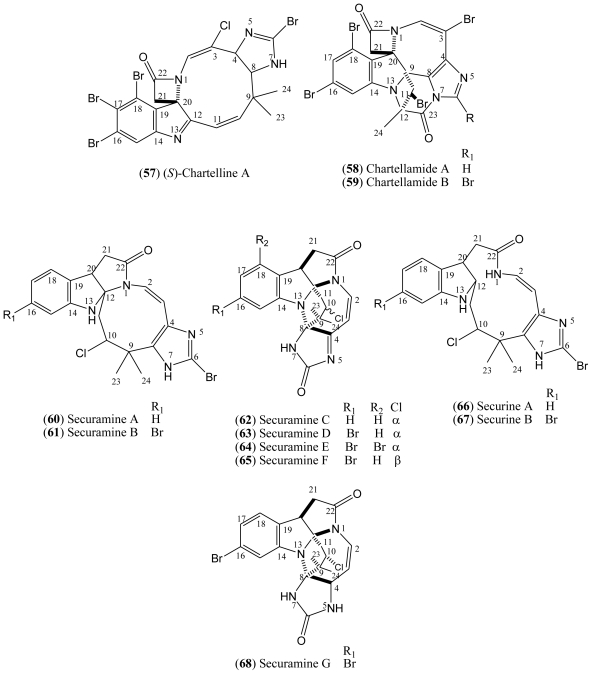
Structures of chartelline, chartellamide, securamines and securines **57**–**68**.

Chartelline A, Chartellamide A, B and C (**57**–**59**, Figure 8) are unusual β-lactam-imidazole alkaloids isolated from the marine bryozoans *Chartella papyrace*a (Flustridae) [56,57]. In addition, other halogenated indole-imidazole alkaloids named securamines were isolated from *Securiflustra securifrons* (Pallas), another member of this family. The halogenated securamines B (**61**) and C (**62**) only differ from securamines A (**60**) and D (**63**), respectively by the presence of a bromine substituent in the benzene ring [58]. Securamines E (**64**), F (**65**) and G (**68**) were isolated from the same bryozoans *S. securifrons* (Pallas) [59]. Securine A (**66**) and B (**67**) were obtained by dissolving securamine A (**60**) and B (**61**), respectively, in DMSO-*d*_6_ [59].

### 1.10. Structural Elucidation

This section reports a compilation of the ^13^C chemical shifts of the halogenated marine indole alkaloids derivatives, meridianins (**5**–**9**), psammopemmins (**30** and **32**), aplicyanins (**33**–**38**), aplysinopsins (**40**–**41**, **43**–**44**, **46**–**51**, **53**), and leptoclinidamines (**56**), which have in common the presence of a 3-substituted indole nucleus. Additionally, the ^13^C data of **10** and **39** are presented for comparison of the ^13^C chemical shifts with halogenated examples. The literature data are listed in Tables 5, 6, 7 and 8. The solvent (A = DMSO-*d_6_*, B = CD_3_OD, and C = CDCl_3_) and references are shown in the first line of the tables.

Inspection of the ^13^C-NMR data of compounds **5**, **8**, **30** and **32** as compared with **10** (Table 5) reveals that introduction of a hydroxyl group in the C-4 indole moiety results in downfield signals at the α carbon. Additionally, comparison of the ^13^C data of meridianin G (**10**), which bears only a 3-substituted indole core, with the other bromine indole derivatives shows that introduction of a bromine in the indole skeleton results in upfield signals at the α carbon.

The meridianin family skeleton can be recognized by the typical ^1^H-NMR signals, as for example, in the case of compound **10**: a pair of doublet for the pyrimidine protons (δ 7.02 and 8.05, *J* = 5.5 Hz), together with a singlet for H-2, the typical pattern of a 3-substituted indole nucleus. The ^13^C-NMR downfield signals at δ C-2′, C-4′, and CH-6′ corroborate the presence of 2-aminopyrimidine at C-3 in compounds **5**–**10** [60].

The basic difference between the psammopemmins and the meridianins is the presence of a 5′-substituted 4′-amino-2′-bromopyrimidine at C-3 of the indole nucleus. The distinguishing ^1^H-NMR signals of the heterocyclic ring of the psammopemmins class can be recognized by the signals at δ 7.12 (d, *J* = 5.4 Hz) and 8.12 (br d, *J* = 5.4 Hz), attributed to the pyrimidine proton H-6′ and to NH at position 1′, as in the case of compound **30**. The ^13^C-NMR downfield signals at δ C-2′, C-4′, and C-5′confirm the presence of 5′-substituted 4′-amino-2′-bromopyrimidine [28].

The aplicianins’ ^13^C-NMR spectra differ from those of the meridianins and psammopemmins because of the presence of the signals due to a guanidine group at low field (C-2′) and three chemicals shifts at upfield, ascribed to C-4′ (CH), C-5′ (CH_2_), and C-6′ (CH_2_). Additionally, the ^1^H-NMR coupling constants of the 6-tetrahydropyrimidine protons are important to establish the difference between aplicyanins, meridianins, and psammopemmins [37].

Aplysinopsins (**39**–**41**, **43**–**44**), with the iminoimidazolidinone substituted at the C-3 of the indole core, normally show a ^1^H-NMR spectrum with signals due to *N*-methyl groups in the range of δ 3.0 to 3.5 (s, 3H), as well as a singlet characteristic of an olefinic proton in the δ 6.38–6.46 range. The ^13^C NMR spectrum reveals the signals for two olefinic carbons C-8 (CH) and C-1′ (C), methyl, guanidine, and amide carbonyl, as well as those of the indole ring, as already mentioned [43,47,48]. Analysis of the ^13^C data of **39**, which bears a 3-substituted indole core, and comparison with data of the other bromine indole derivatives show that the presence of bromine in the indole moiety results in upfield signals at the carbon α.

The spectra of aplysinopsins (**50**–**51**) differ in terms of the signals at C-8, C-3′, and C-5′, if compared with data for 3-iminoimidazolidinone, where C=NH (C-3′) is replaced by C=O (C-3′) [41].

The *E* or *Z*-configuration of the double bond at C-8 could be assigned on the basis of a ^1^H, ^13^C heteronuclear coupling constant. The coupling constant value obtained for the *E* isomer was larger than in the *Z* [41,42]. The geometry of the C-8-C-1′ olefin could be determined by comparison of the chemical shift of the H-2 proton and C-8 carbon. In the *Z* isomer, the δ values of C-8 and H-2 were upfield compared to the values obtained for the *E* isomer [41,42,47]. Aplysinopsin type compounds without substituents at N-2′ are predominantly of *Z* configuration, whereas the converse is true for compounds bearing a methyl group at N-2′. Although it is important to note that, *Z* and *E* aplysinopsin alkaloids undergo rapid isomerization [41,42,61].

Comparison of the ^13^C-NMR data of **46**–**49** with previous aplysinopsins reveals that the C-8 and C-1′ signals are shifted upfield according to R_1_ at C-1′, thereby confirming that the double bond at C-8-C-1′ is absent. Segraves and Crews considered that **48** and **49** are artifacts formed from **47** during the extraction process [48].

The ^13^C-NMR data of compound **53** indicates the presence of two indoles and two iminoimidazolidinones. Biogenetically, this compound could be formed from an enzymatic Diels–Alder cycloaddition of two molecules of aplysinopsin, which were probably derived from tryptophan and guanidine, followed by some modifications [44].

The structure of leptoclinidamine C (**56**) has been established as a 3,6-disubstituted indole and a β-substituted alanine by 1D and 2D NMR data. The ^13^C data indicate the presence of two *N*-methyl groups at C-14 and C-16; a third methyl group at C-13 is attributable to an S-methyl. As mentioned, the chemical shift of the quaternary carbon C-6 (δ 114.6) indicates that the bromine was substituted at this position [55].

## 2. Conclusions

In recent decades the number of new isolated natural compounds, many of which contain halogen, has increased significantly as a consequence of improved collection methods (scuba diving and remote submersibles for accessing deep water organisms), selective bioassays, new separation and purification techniques, and powerful identification methods such as multi-dimensional NMR spectroscopy, high-resolution mass spectrometry, and X-ray diffraction [8,3]. The assignment of carbon signals of a given isolated compound by comparison with the data of known compounds is an important tool for the discovery of novel natural compounds, when the ^13^C-NMR data of appropriate model compounds are available. This was the case with meridianins A–E, which were deduced by 2D NMR spectroscopic methods in combination with comparison to literature data reported for the related natural products the psammopemmins. The indole alkaloids are a class of marine natural products displaying unique promising properties for the development of new drug leads, and they are a wonderful challenge to synthetic chemists. The majority of marine indole alkaloids are rather simple compounds. However, some of the indole alkaloids carry unique structural features. Bacteria and algae have yielded simple halogenated indoles, while more complicated structures have been isolated from marine sources [9]. Over the past 5 years there has clearly been an increasing interest in the isolation, determination of the biological and ecological significance, and synthesis of meridianins, aplysinopsis, and analogues, as confirmed by number of articles and reviews about these marine natural molecules [21,39,63]. Among the different classes of compounds reported here, the protein kinase inhibitors meridianins deserve prominence. Along with variolins, these compounds have inspired the design of the synthetic hybrid meriolins, which constitute a new CDK inhibitory scaffold with promising antitumor activity. On the other hand, aplycianins because of their pronounced antimitotic and cytotoxic potential, have been considered a novel model for anticancer drug discovery. Unfortunately, the biological potential of psammopemmins and the recently isolated leptolinidamines are unknown so far. Finally, aplysinopsins show specific toxicity for cancer cells; however, the most potent pharmacological activity of aplysinopsins is related to modulation of the central nervous system. An interesting fact in all these types of indole skeletons covered here is that halogenations generally occur at C-5, sometimes at C-6, or at both C-5 and C-6 of the indole ring. The bromination of many of the mentioned natural products could be associated with increased biological activity [9].

## Figures and Tables

**Table 1:** Effects of meridianins A–G (**4–10**) on the activity of protein kinases (IC_50_ in μM).

Protein kinase	Meridianins						
	A	B	C	D	E	F	G
CDK1/cyclin B	2.50	1.50	3.00	13.00	0.18	20.00	150.00
CDK5/p25	3.00	1.00	6.00	5.50	0.15	20.00	140.00
PKA	11.00	0.21	0.70	1.00	0.09	3.20	120.00
PKG	200.0	1.00	0.40	0.80	0.60	0.60	400.00
GSK-3β	1.30	0.50	2.00	2.50	2.50	2.00	350.00
CK1	nt 1	1.00	30.00	100.00	0.40	nt 1	nt 1

1 nt: not tested.

**Table 2:** Effects of variolin B (**12**) and meriolins 1(**16**), 10 (**25**), and 11 (**26**) on the activity of protein kinases (IC_50_ in μM).

Protein kinase	Variolin B	Meriolin 1	Meriolin 10	Meriolin 11
CDK1/cyclin B	0.06	0.78	0.24	2.20
CDK2/cyclin A	0.08	0.09	0.06	1.3
CDK5/p25	0.09	0.51	0.23	0.68
CDK9/cyclin T	0.026	0.026	0.05	1.00
GSK-3α/β	0.07	0.63	2.00	30.0
CK1	0.005	0.2	3.0	1.3
DYRK1A	0.08	0.13	0.13	0.3

**Table 3:** Cytotoxicity (GI_50_ values reported in μM) and antimitotic activity (IC_50_, mM) of aplicyanins B (**34**), D (**36**), E (**37**), F (**38**) and (±) aplicyanin A, B, and E.

Compound	Cell lines			Antimitotic Activity
	A-549	HT-29	MDA-MB-231	
Aplicyanin B	0.66	0.39	0.42	1.19
Aplicyanin D	0.63	0.33	0.41	1.09
Aplicyanin E	8.70	7.96	7.96	nt^2^
Aplicyanin F	1.31	0.47	0.81	0.18 0.036
(±)-aplicyanin A	0.27	0.11	0.27	nt
(±)-aplicyanin B	0.51	0.33	0.98	nt
(±)-aplicyanin E	na 1	na	10.9	nt

1 na: not active;

2 nt: not tested.

**Table 4:** Comparison of the biological activity of the main natural halogenated indole alkaloids meridianins, psammopemmins, aplicyanins, and aplysinopsins.

Compound	Biological Activity
**Meridianins**	
Meridianin B (**5**)	Inhibition of protein kinases; Cytotoxicity [14,15]
Meridianin C (**6**)	Inhibition of protein kinases; Cytotoxicity [14,15]
Meridianin D (**7**)	Inhibition of protein kinases; Cytotoxicity [14,15]
Meridianin E (**8**)	Inhibition of protein kinases; Cytotoxicity [14,15]
Meridianin F (**9**)	Inhibition of protein kinases [14]

**Psammopemmins**	
Psammopemmin B (**31**)	nt 1
Psammopemmin C (**32**)	nt

**Aplicyanins**	
Aplicyanin A (**33**)	na 2
Aplicyanin B (**34**)	Cytotoxicity and antimitotic activity [37]
Aplicyanin C (**35**)	na
Aplicyanin D (**36**)	Cytotoxicity and antimitotic activity [37]
Aplicyanin E (**37**)	Cytotoxicity [37]
Aplicyanin F (**38**)	Cytotoxicity and antimitotic activity [37]

**Aplysinopsins**	
6-bromo-2′-de-N-methylaplysinopsin (**40**)	Antimalarial [46]; Serotonin receptors modulator [46]
	Inhibitor of nitric oxide synthase (nNOS) [47]
6-bromoaplysinopsin (**41**)	Antimalarial [46]; Serotonin receptors modulator [46]
	Allelochemical [54]
6-bromo-4′-de-*N*-methylaplysinopsin (**42**)	nt
6-bromo-4′-demethyl-3′-*N*-methylaplysinopsin (**43**)	nt
5,6-dibromo-2′-demethylaplysinopsin (**44**)	Inhibitor of nitric oxide synthase (nNOS) [47]
6-bromo-1′,8-dihydro-aplysinopsin (**46**)	Antimicrobial [48]
6-bromo-1′-hydroxy-1′,8-dihydroaplysinopsin (**47**)	Antimicrobial [48]
6-bromo-1′-methoxy-1′,8-dihydroxyaplysinopsin (**48**)	Antimicrobial [48]
6-bromo-1′-ethoxy-1′,8-dihydroxyaplysinopsin (**49**)	Antimicrobial [48]
6-bromo-3′-deimino-3′-oxoaplysinopsin (**50**)	nt
6-bromo-3′-deimino-2′,4′-bis(demethyl)-3′-Oxoaplysinopsin (**51**)	nt
Dimer of 6-bromo-2′-de-*N*-methylaplysinopsin (**52**)	Antimicrobial [54]
Tubastrindole A (**53**)	-

1 nt: not tested.

2 na: not active.

**Table 5:** ^13^C chemical shifts (δ in ppm) of meridianins, psammopemmins, and aplicyanins halogenated derivatives.

Carbon	5	6	7	8	9	10	30	32	33	34	35	36	37
Solvent	A	A	A	A	B	B	A	A	B	B	B	B	B
Ref.	14	14	14	14	60	60	25	25	37	37	37	37	37
2	129.9	129.6	129.2	129.2	131.6	129.1	128.3	128.8	125.3	125.6	123.8	124.1	124.7
3	113.7	113.3	114.8	116.1	114.9	115.4	113.7	113.6	113.6	113.8	112.0	111.2	112.3
3a	114.0	127.1	124.5	115.2	127.6	126.8	114.3	113.9	124.1	127.8	124.3	124.1	123.5
4	153.0	124.6	124.3	152.0	127.7	122.7	152.0	152.9	121.9	121.8	122.5	122.4	124.6
5	108.8	113.4	123.1	107.3	118.4	123.4	105.4	108.4	115.2	114.3	114.4	114.6	119.3
6	116.7	124.7	113.9	126.7	117.1	121.9	124.3	116.4	125.9	126.1	126.9	127.1	116.4
7	105.3	113.9	114.5	92.6	117.5	112.8	102.3	105.0	114.5	114.6	111.4	111.5	114.6
7a	139.7	135.9	138.0	136.9	138.5	138.9	139.2	139.5	137.2	137.2	132.7	132.7	133.6
2′	160.7	163.6	163.6	160.2	165.3	165.6	161.7	161.6	155.7	152.3	155.7	152.4	155.7
4′	160.8	162.3	162.3	161.8	163.9	165.6	160.7	159.7	48.1	48.2	47.6	47.7	47.4
5′	104.6	105.4	105.4	104.8	107.1	107.6	158.3	158.8	28.3	26.9	28.3	26.6	28.3
6′	157.1	157.2	157.2	159.0	156.2	157.5	104.3	104.3	38.6	38.6	38.4	38.2	38.3
CH_3_CO										173.9		174.0	
CH_3_CO										24.1		24.1	
OCH_3_											66.8	66.7	67.0

**Table 6:** ^13^C chemical shifts (δ in ppm) for halogenated aplicyanin **38** and aplysinopsins derivatives.

Carbon	38	39	40 (Z)	40 (E)	41	43 (Z)	43 (E)	44 (*Z*)	44 (*E*)	50 (E)	51 (Z)	51 (E)
Solvent	B	A	A	A	A	A	A	A	A	A	A	A
Ref.	37	62	42	42	43	42	42	47	47	41	41	41
2	125.0	127.4	129.6	129.0	127.7	129.9	129.0	130.6	132.6	129.3	127.5	130.1
3	111.5	108.4	111.9	110.7	108.7	112.0	110.8	108.3	108.2	108.7	108.7	108.9
3a	123.2	127.8	125.7	126.7	126.9	125.7	126.8	127.8	128.4	127.5	126.3	127.3
4	124.5	118.1	121.0	119.9	119.6	121.1	120.0	123.6	122.5	120.2	120.1	119.2
5	119.5	119.5	122.4	122.5	121.8	122.4	122.5	116.9	116.9	122.6	122.9	122.8
6	116.6	121.8	114.5	114.4	121.8	114.5	114.5	115.2	115.2	114.0	115.0	114.6
7	114.6	111.8	114.3	114.4	114.0	114.3	114.4	116.8	117.2	114.6	114.4	115.0
7a	133.6	135.7	136.7	136.5	136.2	136.5	136.5	135.8	135.8	136.5	136.6	136.5
8		102.8	106.1	113.3	101.5	106.5	113.9	108.9	115.1	107.5	101.0	106.5
1′		126.5	136.9	134.9	126.9	136.9	136.6	135.7	135.7	125.1	124.9	125.4
2′	152.3											
3′	26.8	150.8	157.7	155.0	162.1	157.1	154.7	155.9	152.7	152.8	155.4	153.4
4′	47.6											
5′	26.8	162.3	169.0	167.0	150.4	169.0	167.1	163.2	160.8	162.0	167.3	163.9
6′	38.1											
2′-NCH_3_		27.0			24.4	27.8	27.8			24.3		
4′- NCH_3_		24.9	25.6	25.6	26.6	25.5	25.4	26.0	26.0	26.3		
OCH_3_	67.1											
CH_3_CO	174.0											
CH_3_CO	24.1											

**Table 7:** ^13^C chemical shifts (δ in ppm) for halogenated aplysinopsins and leptoclinidamines derivatives.

Carbon	46	47	48	49	53	56	Carbon	46	47	48	49	53	56
Solvent	B	B	B	B	ni 1	A	4′					119.7	
Ref.	48	48	48	48	44	55							
2	124.8	125.1	125.4	125.4	123.3	129.0	5′	171.8	171.9	169.7	170.0	124.8	
3	106.5	105.3	104.6	104.7	115.0	109.4	6′					117.3	
3a	125.7	125.6	125.6	125.6	126.4	124.9	7′					115.9	
4	119.3	119.3	119.4	119.0	120.1	122.9	7a′					137.4	
5	121.9	122.0	122.1	122.0	121.5	123.4	8′					44.5	
6	114.7	114.8	114.9	114.9	125.9	114.6	9′					72.3	
7	113.9	114.0	114.0	114.0	113.0	114.8	11′					159.0	
7a	137.2	137.1	137.2	137.2	139.5	137.0	13′					172.5	
8	29.4	30.1	30.1	30.4	27.6	164.1	2′-NCH_3_	24.7	25.3	25.6	25.6		
9					72.3		4′- NCH_3_	24.1	24.6	24.7	24.6		
10						50.0	10-NCH_3_					33.1	
11					161.6	25.7	12-NCH_3_					26.6	
12						136.4	13-SCH_3_						18.6
13					174.0	125.9	14-NCH_3_						33.5
15						138.1	16-NCH_3_						34.2
17						172.2	10′-NCH_3_					28.4	
1′	64.0	89.0	94.3	93.8			12′-NCH_3_					26.5	
2′					125.6		OCH_3_			52.3			
3′	158.1	156.6	157.2	157.0	104.4		OCH_2_				61.5		
3′a					127.7		CH_3_				13.7		

1 ni: not informed.

**Table 8:** ^13^C chemical shifts (δ in ppm) for halogenated chartelline, chartellamides, securamines and securines derivatives.

Carbon	58	59	60	61	62	63	64	65	66	67	68
Solvent	C	C	C	C	C	C	C	C	A	A	C
Ref.	57	57	58	58	58	58	59	59	58	58	59
2	120.4	120.6	127.4	127.1	135.9	136.1	136.4	135.9	130.2	130.3	48.3
3	109.5	108.1	95.1	95.3	101.6	101.2	101.2	101.5	100.9	103.6	96.3
4	130.5	130.8	115.8	116.0	187.5	188.0	187.3	187.2	121.1	121.5	140.7
6	133.2	116.6	122.5	121.1	166.6	166.7	166.4	166.0	125.0	127.6	156.8
8	126.6	129.5	145.5	144.8	85.6	85.7	85.4	84.7	135.6	135.3	80.7
9	65.9	65.2	41.6	41.9	44.0	43.9	44.2	43.7	40.6	40.8	39.2
10	41.6	41.6	64.9	64.6	59.4	59.5	58.7	52.1	71.2	70.9	61.4
11	45.9	46.8	48.6	48.3	41.8	41.7	41.9	42.8	30.8	30.8	41.0
12	67.9	70.5	87.4	87.4	89.2	89.1	88.0	89.8	132.7	133.9	87.9
14	141.5	141.1	147.0	148.0	147.0	145.7	148.0	146.9	134.4	134.5	147.5
15	117.6	117.6	109.3	112.2	114.7	111.1	113.5	114.7	110.9	113.2	114.6
16	125.9	126.0	129.0	122.3	123.1	129.4	120.6	123.0	120.5	114.1	122.3
17	132.5	132.5	119.9	122.5	124.9	121.9	128.0	124.9	118.1	119.4	123.5
18	119.4	119.4	123.9	125.0	125.6	124.6	123.8	125.5	117.5	121.1	125.2
19	124.8	124.7	127.8	126.7	128.0	128.8	126.3	127.9	128.5	126.0	130.4
20	67.4	67.6	50.0	49.4	45.0	45.3	46.8	44.9	105.7	106.3	45.6
21	47.8	48.0	34.1	33.8	34.2	34.4	32.6	34.2	30.7	30.6	33.8
22	162.5	162.1	172.8	172.2	170.3	170.5	170.0	170.0	169.4	169.1	171.2
23	164.9	164.1	19.0	18.9	17.2	17.3	17.1	18.6	19.8	19.8	15.4
24	15.4	17.1	31.9	31.8	21.1	21.1	21.1	22.6	28.7	28.7	21.1
